# Insights into the dynamic nature of the dsRNA-bound TLR3 complex

**DOI:** 10.1038/s41598-019-39984-8

**Published:** 2019-03-06

**Authors:** Vijayakumar Gosu, Seungwoo Son, Donghyun Shin, Ki-Duk Song

**Affiliations:** 1 Department of Animal Biotechnology, Chonbuk National University, Jeonju, 54896 Republic of Korea; 2 The Animal Molecular Genetics and Breeding Center, Chonbuk National University, Jeonju, 54896 Republic of Korea

## Abstract

Toll-like receptor 3 (TLR3), an endosomal receptor crucial for immune responses upon viral invasion. The TLR3 ectodomain (ECD) is responsible for double-stranded RNA (dsRNA) recognition and mutational analysis suggested that TLR3 ECD C-terminal dimerization is essential for dsRNA binding. Moreover, the L412F polymorphism of TLR3 is associated with human diseases. Although the mouse structure of the TLR3-dsRNA complex provides valuable insights, the structural dynamic behavior of the TLR3-dsRNA complex in humans is not completely understood. Hence, in this study, we performed molecular dynamic simulations of human wild-type and mutant TLR3 complexes. Our results suggested that apoTLR3 ECD dimers are unlikely to be stable due to the distance between the monomers are largely varied during simulations. The observed interaction energies and hydrogen bonds in dsRNA-bound TLR3 wild-type and mutant complexes indicate the presence of a weak dimer interface at the TLR3 ECD C-terminal site, which is required for effective dsRNA binding. The L412F mutant exhibited similar dominant motion compared to wild-type. Additionally, we identified the distribution of crucial residues for signal propagation in TLR3-dsRNA complex through the evaluation of residue betweenness centrality (C_B_). The results of this study extend our understanding of TLR3-dsRNA complex, which may assist in TLR3 therapeutics.

## Introduction

The innate immune system is crucial for host defense against pathogenic invasion^1,2^. The innate immune response is dependent on pattern recognition receptors, which trigger conserved host defense signaling pathways^3^. Among several pattern recognition receptors, Toll-like receptors (TLRs) are crucial for immune response^4^. TLRs are highly conserved transmembrane receptors expressed on the cell surface and endosomes; they recognize a conserved molecular pattern from microbial pathogens^5^. TLR3, a well-studied TLR, is localized on endosomes and detects double-stranded RNA (dsRNA) released from viruses during invasion or necrotic cells during inflammation^6^. Upon sensing dsRNA, TLR3 recruits the adaptor TIR-domain-containing adapter-inducing interferon-β (TRIF) via toll/interleukin (IL)-1 receptor (TIR)-TIR domain interactions in the cytoplasm. TRIF, in turn, recruits receptor-interacting protein 1 (RIP1) to activate nuclear factor-κB (NF-κB) via TNF receptor-associated factors (TRAFs), and the IκB kinase (IKK) complex associates with signaling cascades, resulting in the regulation of immune responses against many viruses^5^. However, unregulated or uninterrupted TLR3-mediated immune responses may have severe consequences, including death, in some viral infection models^7^. TLR3 deficiency may also increase the risk of herpes simplex encephalitis and coxsackievirus infection^8,9^. Moreover, a recent study has reported several novel mutations in TLR3 signaling pathway molecules that are associated with impaired innate immunity and an increased susceptibility to herpes simplex encephalitis^10^. In addition, defects in TLR3 signaling increase susceptibility to chikungunya virus infection^11^. Single nucleotide polymorphisms (SNPs) in TLR genes are likely to influence the structure and functional relationships among these TLRs and are associated with a wide range of diseases^12^. In particular, in *TLR3*, a wide range of SNPs have been identified, which are associated with several diseases^13–19^. The TLR3 L412F polymorphism is associated with cytomegalovirus infection in children^13^, as well as with reduced natural killer cell responsiveness with susceptibility to recurrent herpes labialis^18^. *TLR3* polymorphisms are also associated with several diseases, including nasopharyngeal carcinoma in the Cantonese population^20^, age-related macular degeneration^15^, oral cancer^16^, and HCV infection^14^.

**Figure 1:**
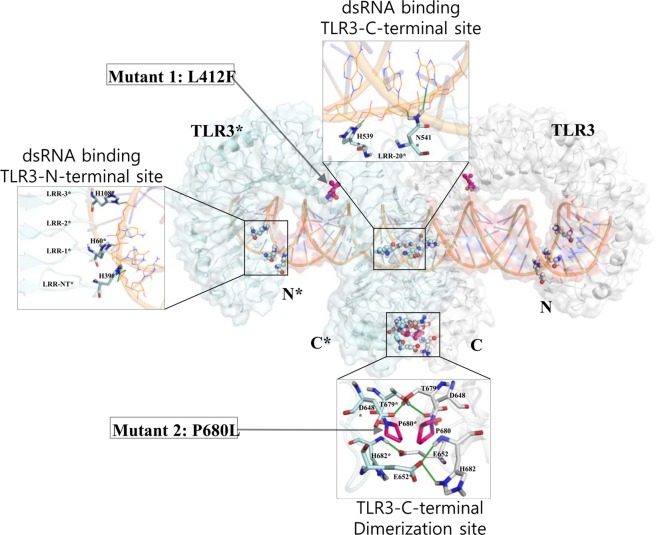
The structure of dsRNA-bound TLR3 complex. The structure of the human TLR3-dsRNA complex is shown. Individual chains of the TLR3 complex are shown in white (TLR3) and pale cyan (TLR3*). dsRNA is shown in orange. The three crucial interaction sites are also shown. Hydrogen bonds identified using PyMOL shown in green. For clarity, only the interactions from TLR3* (chain B) in the N- and C-terminal interaction sites of dsRNA (46 bp) are shown. The mutant residues are shown in magenta. Mutant 1, L412F, is a naturally-occurring polymorphism in human population, whereas mutant 2, P680L, is a point mutation considered for this study.

TLR3 is composed of an extracellular domain (ECD) at the cell surface, a single transmembrane domain, and an intracellular TIR domain (all TLRs share this common domain architecture). The human TLR3 ECD includes 23 leucine-rich repeats (LRR) of ~24 aa, which in turn form an α-helix and β-strand connected by a loop, thus forming a horseshoe-shaped solenoid structure. The N- and C-terminal regions of TLR have special structures called the LRR-NT and LRR-CT^21^. The TLR3 ECD detects dsRNA, resulting in TLR3 homo-dimerization via the TIR-TIR domain and signal transduction by recruiting and interacting with adaptor molecules at the intracellular level. The dsRNAs of longer than 30 bp are candidates to induce innate immune responses to curb viral infection^22^ and protein crystallography studies have shown that mouse TLR3 binds to 46-bp dsRNA^23^. The mouse TLR3-dsRNA complex (PDB ID: 3CIY) shows two interaction sites for dsRNA located on the lateral convex surface at the N- and C-terminal regions of the TLR3 ECD and a single TLR3 dimer interface at the C-terminal site. The TLR3 sequence identity between mice and humans is of 78.7%, wherein the number of most of the interacting residue numbers is shared. The N-terminal interaction site includes the LRR-NT and 1–3 LRR components, consisting of His39, His60, Gln62, Arg64, Phe84, His108, Glu110, and Ser112 (identical interacting residues from mice and humans are given). The critical residues for interactions, His39, His60, and His108, are highly conserved among species. The C-terminal site includes 19–21 LRR components consisting of Asn515, Asn517, His539, Asn541, Arg544, and Ser571. Moreover, two TLR3 ECDs form the homodimer interface at the LRR-CT via Asp648, Glu652, Thr679, Pro680, and His682^23^. Further mutational analysis of human TLR3 has revealed that His39, His60, His108, His539, and Asn541 residues interact with dsRNA, and the C-terminal dimerization site is critical for dsRNA binding as well as TLR3 signaling^24^ (Fig. 1).

The dsRNA-unbound ECD has been solved for TLR3^21^, but it is still unclear whether endosomal nucleic acid-sensing TLRs exist as monomers or dimers. A previous study of human TLR9 indicated that TLR9 exists as a preformed dimer^25^. A mutational analysis has shown that the TLR3 ECD C-terminal dimer interface is required for the binding of dsRNA to TLR3. In particular, it has been stated that the P680 mutation to leucine in the TLR3 ECD C-terminal site reduces the binding affinity of dsRNA to TLR3 and affects subsequent signaling^24^. The mouse TLR3-dsRNA complex solved by crystallography has provided structural information for TLR3 dimer formation, the orientation of individual chains, and dsRNA-induced conformational changes on TLR3. However, understanding the dynamic behavior of the TLR3-dsRNA complex is crucial to gain insights into the regulatory mechanisms of TLR3 dimerization. In addition, studies on the importance of the TLR3 C-terminal dimerization site, as well as on the cooperative binding of dsRNA to TLR3 at three different interaction sites in terms of TLR3 dynamics, may assist in TLR3 therapeutics. Furthermore, it is also essential to understand the impact of L412F, which is the natural variant of TLR3, on the structure-function relationship, which is particularly important owing to its association with several diseases^13,17,18^.

To address the abovementioned issues, we prepared dsRNA-bound TLR3 wild-type monomer (mTLR3^WT^-dsRNA), dsRNA-unbound TLR3 wild-type dimer (apo_dTLR3^WT^), dsRNA-bound TLR3 wild-type dimer (dTLR3^WT^-dsRNA), dsRNA-bound TLR3 dimer with a mutation at position 412 (dTLR3^L412F^-dsRNA), and dsRNA-bound TLR3 dimer with a mutation at position 680 (dTLR3^P680L^-dsRNA) complex systems for the human TLR3 ECD. For simplicity, hereafter we refer to these complexes as mTLR3^WT^-dsRNA, apo_dTLR3^WT^, dTLR3^WT^-dsRNA, dTLR3^L412F^-dsRNA, and dTLR3^P680L^-dsRNA wherever necessary. We investigated the dynamic behavior of all five TLR3 complexes using molecular dynamic (MD) simulations to elucidate the initial mechanism of dsRNA binding to TLR3 at the extracellular surface. Computational approaches and MD simulations are often used to determine protein structure-function relationships via ligand- and mutation-induced conformational changes^26,27^. Subsequently, we employed a principal component analysis (PCA) and residue network centrality analysis^28^ to investigate the global motions and the distribution of crucial residues for signal transmission within the TLR3 complexes, respectively.

## Materials and Methods

### TLR3-dsRNA complex preparation

The human TLR3 sequence of the ECD was obtained from UniProtKB (UniProt ID: O15455). Although the structure of human TLR3 is already available in the Protein Data Bank (PDB) as a monomer (PDB ID: 2A0Z), to achieve the accurate TLR3 dimer conformation, a TLR3 dimer model was built by homology modeling using the mouse TLR3 dimer crystal structure (PDB ID: 3CIY) as a template. The sequence identity and structural root mean square deviation (RMSD) between mouse and human TLR3 ECD were 78.7% and 1.36 Å, respectively. The TLR3 sequence was submitted to the Swiss-Model web server^29^ using default parameters to construct the TLR3 dimer model. The stereochemical properties were checked using the ProQ web server (http://proq.bioinfo.se/ProQ/). The TLR3 dimer was included as the apo_dTLR3^WT^ model. Furthermore, to construct the dTLR3^WT^-dsRNA complex, the apo_dTLR3^WT^ model was superimposed on the mouse TLR3-dsRNA complex (PDB ID: 3CIY) and the dsRNA was retrieved. The dsRNA-bound TLR3 monomer (mTLR3^WT^-dsRNA) was built by removing one TLR3 monomer from the dTLR3^WT^-dsRNA complex. To construct the mutational complexes (dTLR3^L412F^-dsRNA and dTLR3^P680L^-dsRNA), Discovery Studio Visualizer (Discovery Studio 2.1; Accelrys Inc., San Diego, CA, USA) was used for the computational mutation by substituting F at the 412^th^ position and L at the 680^th^ position with probable rotamers in the dTLR3^WT^-dsRNA complex. Subsequently, the stereochemical properties of all five complexes, mTLR3^WT^-dsRNA, apo_dTLR3^WT^, dTLR3^WT^-dsRNA, dTLR3^L412F^-dsRNA, and dTLR3^P680L^-dsRNA, were analyzed. Since TLR3 ECD may undergo minute conformational changes upon dsRNA binding, for comparison we have constructed another model of the apo_dTLR3^WT^ complex using the human TLR3 monomer crystal structure (PDB ID: 2A0Z) by superposing it onto the dsRNA-bound mouse TLR3 crystal structure (PDB ID: 3CIY). We removed the steric clashes/bumps at TLR3 ECD C-terminal dimerization site.

### Molecular dynamic (MD) simulations

Since dsRNA binds to TLR3 at a slightly acidic pH (5.5 to 6.5), we examined the possible protonation states for charged residues of TLR3 at pH 6.0 using the H++ web server^30^, particularly for histidine residues owing to their interaction with the dsRNA backbone. Subsequently, explicit MD simulations were performed for all of the TLR3 complex systems using Gromacs 5.1.4^31,32^, as reported in our previous studies^33^. AMBER-ff99SB-ILDN force field^34^ (this force field contains the parameters for both protein and nucleic acids, hence we used this to simulate both protein and dsRNA) and tip3p water molecules were used to generate the solvated system and a triclinic box was used with periodic boundary conditions at a distance of 1.6 nm. Appropriate counter ions were included to neutralize the system. Energy minimization was performed using the steepest descent method with a maximum tolerance of 1000 kJ/mol/nm. The Particle Mesh Ewald (PME) approach was employed for long range electrostatics with a 1.0-nm cutoff for short range electrostatics as well as Vander walls interactions. PME grid spacing of 0.16 nm was used for FFT with fourth-order cubic interpolation. Bonds were constrained using the LINCS algorithm^35^. Constant volume (NVT) ensemble simulations were performed for 0.1 ns; subsequently, simulations were performed in the constant pressure (NPT) ensemble for 0.5 ns for each system using positional restraints. A modified Berendsen thermostat (v-rescale)^36^ and Parrinello-Rahman barostat^37^ were used to maintain the temperature (300 K) and pressure (1.0 bar), respectively. Finally, production simulations were performed in the absence of any positional restraints for 100 ns. A total of three independent simulation runs with different initial velocities were generated for all five complexes. Additionally, 100 ns simulations were employed for the dTLR3^WT^-dsRNA complex at pH 5.0 to evaluate the dynamic behavior of the dsRNA-bound TLR3^WT^ complex at a more acidic pH. We also employed 50 ns simulations for the apo_dTLR3WT complex built using a human TLR3 monomer structure. The 2-ps coordinates were saved for the whole trajectory by applying a 2-fs time step and analysis was carried out using the 20- or 10-ps coordinates. Most of the analyses were performed using gromacs analysis tools and all plots were generated using Excel.

### Interaction energy

Interaction energies between various components within TLR3 complexes of all the systems were calculated using the rerun option in the gmx mdrun module for the whole MD trajectory.

### Principal component analysis (PCA)

To determine the dominant modes from the MD trajectories of all systems, PCA was used according to the methods described in previous reports^33^. Briefly, using 10-ps coordinates from last 60 ns of 3 simulation runs from each complex were concatenated and subjected to analysis and overall rotational and translational movements were removed. The gmx covar and gmx anaeig tool was used to obtain the eigenvalues and eigenvectors and to analyze the data^38,39^. Free energy landscape analysis were performed using the gmx sham tool in the gromacs package. FEL plots were drawn using Mathematica 11.2.0 trail version with the help of the script (xpmtotxt.py) from http://nmr.chem.uu.nl/adrien/course/molmod/analysis2.html.

### Network centrality

We constructed a weighted graph residue-residue interaction network, where each amino acid residue represents the node and where weight represents the number of hydrogen bonds between the residues. In our network model, we provided a contact distance less than or equal to 7 Å (non-hydrogen atoms) between two residues. This cut-off distance is used in the literature^28^, hence we applied this in our study. Furthermore, we calculated the betweenness centrality to identify the central node, which is crucial for signal transduction within the protein. We have used the Brandes algorithm^40^ and computed residue betweenness centrality as reported in the literature^28^

$${C}_{B}(v)=\frac{2}{(N-2)(N-1)}\sum _{s=1}^{N-1}\sum _{t=s+1}^{N}\frac{{\sigma }_{st}(v)}{{\sigma }_{st}}$$

where *σ*_*st*_ is the number of shortest paths connecting the nodes *s* and *t*, and *σ*_*st*_ (*v*) is the number of shortest paths connecting the nodes *s* and *t* through the node *v*; $\frac{2}{(N-2)(N-1)}$ is the normalization constant.

## Results

### Structural stability of the TLR3-dsRNA complex

**Figure 2:**
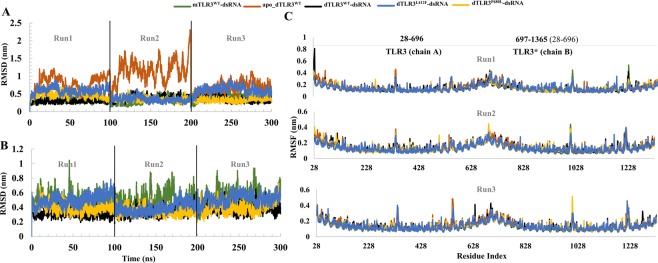
Structural deviations and residue flexibility of TLR3 complexes. (**A**) Root mean square deviation (RMSD) of backbone atoms of all the TLR3 complexes. (**B**) RMSD of dsRNA. (**C**) Root mean square fluctuations (RMSF) of backbone atoms of individual chains of TLR3 for the last 60 ns of the MD simulations. TLR3* starts from 28 and ends at 696, however we used the continuous number for simulations (i.e. 697–1365).

We first prepared five complex systems (mTLR3^WT^-dsRNA, apo_dTLR3^WT^, dTLR3^WT^-dsRNA, dTLR3^L412F^-dsRNA, and dTLR3^P680L^-dsRNA) and subjected each to three independent MD simulation runs of 100 ns (total 300 ns) using different initial velocities. To assess the stability of the simulations, we calculated the protein backbone RMSD. The RMSD plot strongly indicated that except for apo_dTLR3^WT^, the other four systems converged in the final 60 ns (Fig. 2A). Predictably, the apo TLR3 dimer (apo_dTLR3^WT^) showed large fluctuation and variation in 3 runs, indicating that the pre-formed TLR3 ECD dimer may not be stable in physiological conditions. In addition, to verify the modelling artifacts in apo TLR3 dimer complex, additional 50 ns simulation of apo_dTLR3^WT^ built using the human TLR3 monomer structure were also performed, however this complex also exhibits large fluctuations during simulations (Supplementary Fig. S1). Several reports have suggested that ligand-induced dimerization occurs for TLRs^41^, and it is also worth mentioning that human TLR3 is expressed in solution as a monomer^21^. Furthermore, dsRNA-bound TLR3 wild-type complexes (mTLR3^WT^-dsRNA and dTLR3^WT^-dsRNA) showed stable deviations compared to TLR3 mutants. In particular, the dTLR3^L412F^-dsRNA complex showed large deviations (Fig. 2A), indicating that mutations in TLR3 may lead to conformational changes within the protein. The RMSD of the dsRNA showed similar deviations in all of the complexes, except in mTLR3^WT^-dsRNA, which consists of only one monomer that can stabilize one end of the dsRNA, while the other end of the dsRNA is highly flexible. The stable deviation of dsRNA within dTLR3^WT^-dsRNA as well as in mutant complexes (dTLR3^L412F^-dsRNA and dTLR3^P680L^-dsRNA) strongly suggests that both ends of dsRNA were stabilized by cooperative binding via the counter TLR3 monomer (Fig. 2B). To verify the residue fluctuations during simulation, we calculated the root mean square fluctuations (RMSF) of protein backbone atoms of all of the complexes for the last 60 ns. In the dsRNA-unbound (apo_dTLR3^WT^) complex, overall both chains showed larger fluctuations than those of the dsRNA-bound complexes. The individual chains of the dsRNA-bound complexes, mTLR3^WT^-dsRNA, dTLR3^WT^-dsRNA, dTLR3^L412F^-dsRNA, and dTLR3^P680L^-dsRNA, showed stable fluctuations during the simulations, suggesting that dsRNA binding to TLR3 stabilizes the amino acids at the N-terminal and C-terminal sites, which is the only reported segment involved in interactions between dsRNA and TLR3 (Fig. 2C). However, first few residues at the N-terminal show large flexibility particularly in dTLR3^WT^-dsRNA complex (chain A) in simulation run1. Additionally, it is worth noting that all of the complexes showed large fluctuations at LRR12 (323–355) and LRR20 (531–562). In particular, LRR12 contained a long flexible loop (335–343) hanging from the lateral face of the ECD, required for the cleavage of TLR3 by cathepsins for conversion to the cleaved or associated form of functional TLR3^42,43^. The flexible loop present in LRR20 was in close proximity to the dsRNA-binding region. Furthermore, based on the identical residues in both chains, we compared the RMSF of one chain to the RMSF of the counter chain. All of the complexes showed a linear RMSF range and similar correlation coefficients (Supplementary Fig. S2). Importantly, the correlation between the RMSF values for the two chains was less in dTLR3^WT^-dsRNA compared to other complexes, this is due to high fluctuations were observed at the N-terminal region for first few residues (particularly in simulation run 1). however, apo_dTLR3^WT^ showed a larger correlation coefficient than that of dsRNA bound complexes (Supplementary Fig. S2).

We also assessed the radius of gyration to evaluate the rigidity and compactness of the complexes during simulations, providing insights into the overall dimension of the proteins and dsRNA. The Rg plots of the TLR3 backbone atoms for all of the complexes indicate that mutant complexes show slightly larger values than those of wild-type complexes. Overall, TLR3 as well as dsRNA were largely compact during simulations (Supplementary Fig. S3) except for apo_dTLR3^WT^ (simulation run 2). In addition, we calculated the SASA for the backbone atoms of all of the complexes and observed that protein exposure was ~600 nm^2^; however, there were no substantial variations in the SASA values between the wild-type and mutant complexes, and mTLR3^WT^-dsRNA showed an exposure area of 300 nm^2^ (Supplementary Fig. S3).

### Hydrogen bond analysis

**Figure 3:**
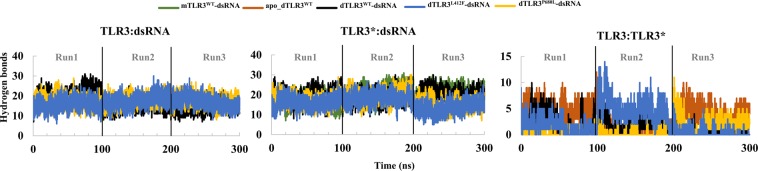
Number of hydrogen bonds between components in TLR3 complexes. Number of hydrogen bonds between TLR3 and dsRNA, as well as between TLR3 and TLR3*, were evaluated with a cut-off distance 0.35 nm and an angle 30°.

To assess the interactions in the TLR3 complex, we analyzed the number of hydrogen bonds (cutoff distance 0.35 nm and angle 30°) between the TLR3 and dsRNA for all of the complexes. We calculated the average hydrogen bonds from the last 60 ns of 3 simulation runs from each complex. For mTLR3^WT^-dsRNA, ~20 hydrogen bonds were observed. In dTLR3^WT^-dsRNA, ~17 and ~20 hydrogen bonds were observed for both of the chains (TLR3 and TLR3*) of TLR3. Although similar hydrogen bonds observed between dsRNA and one TLR3 monomer in mTLR3^WT^-dsRNA (Fig. 3), for the complexation at the TIR domain, counter-TLR3 binding to the other site of dsRNA is required. Moreover, the mutants showed slightly fewer hydrogen bonds compared to the wild type, i.e. ~17 between dsRNA and both of the TLR3 chains (Fig. 3). These results indicate that mutants may induce conformational changes at the surrounding regions of mutant residue which may lead to less contact with dsRNA, thereby resulting in a reduced binding affinity, as reported in a previous study particularly for dTLR3^L412F^-dsRNA^44^. We further analyzed the C-terminal dimer interface and identified, on average, 1, 3, 2, and 1 hydrogen bonds for dTLR3^WT^-dsRNA, apo_dTLR3^WT^, dTLR3^L412F^-dsRNA and dTLR3^P680L^-dsRNA, respectively (Fig. 3). This analysis clearly shows that the C-terminal dimerization interface of TLR3 ECD is required for dsRNA binding. In particular, apo_dTLR3^WT^ maintained ~3 hydrogen bonds throughout the simulations compared to other complexes. However, dTLR3^WT^-dsRNA showed only 1 hydrogen bond, indicating that a weak dimer interface can be maintained upon dsRNA binding to TLR3, thereby stabilizing the whole complex. dTLR3^L412F^-dsRNA showed, on average, ~2 interaction at the C-terminal dimer interface of TLR3 ECD, suggesting that this mutant also maintains a weak C-terminal TLR3 ECD dimer interface, like that of dTLR3^WT^-dsRNA. Furthermore, dTLR3^P680L^-dsRNA showed no interactions at the C-terminal dimer interface in 2 simulation runs, whereas in the third simulation run ~3 interactions on average, suggesting the presence of local conformational changes at the TLR3 ECD C-terminal dimerization interface as shown in previous reports^24^.

### Molecular interactions between dsRNA and TLR3

**Figure 4:**
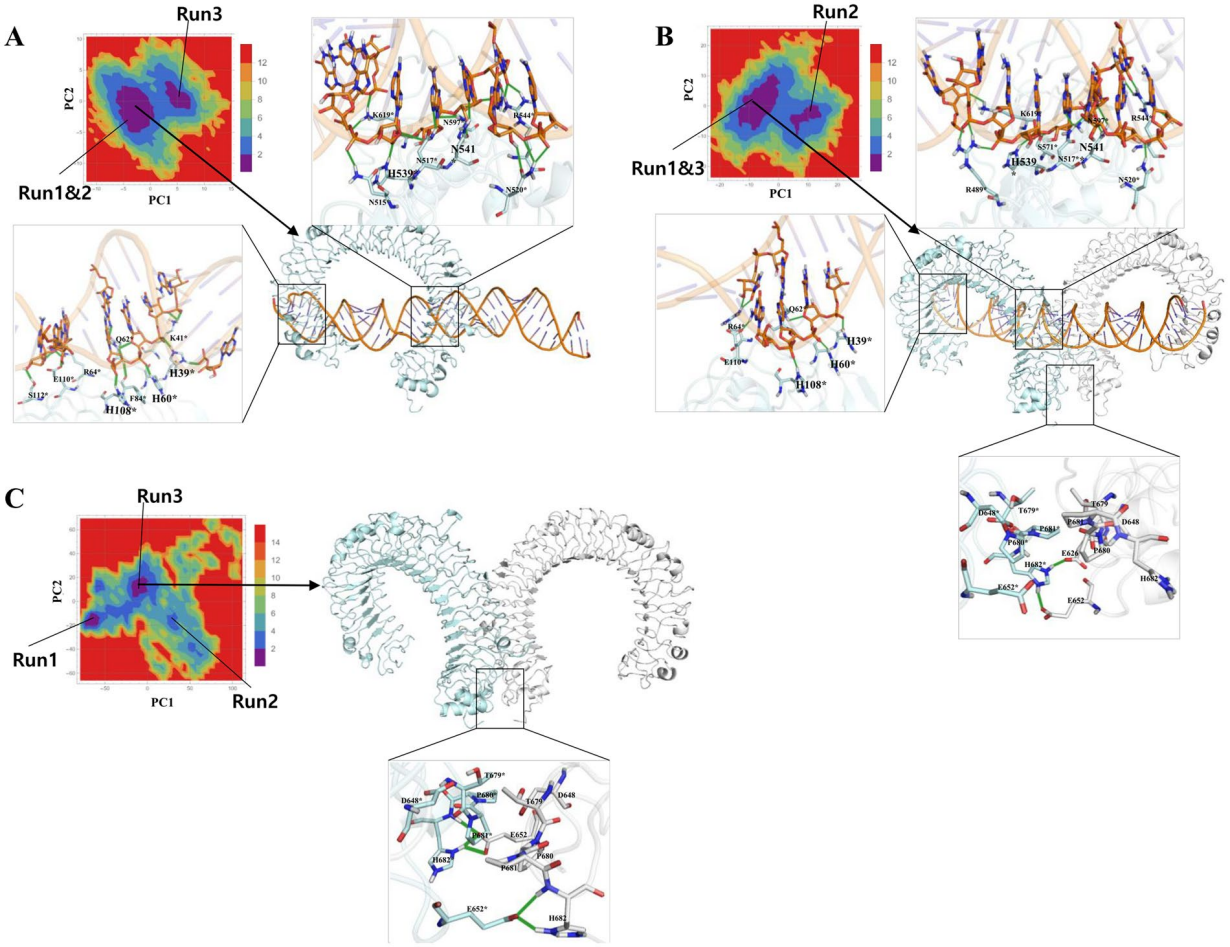
Hydrogen bond interaction between dsRNA and TLR3 wild-type complexes. Hydrogen bond interactions between dsRNA and TLR3 are shown. Representative structures were extracted based on the free-energy landscape of PC1 against PC2. (**A**) mTLR3^WT^-dsRNA. (**B**) dTLR3^WT^-dsRNA. (**C**) apo_dTLR3^WT^. Hydrogen bond interactions between dsRNA and TLR3* (chain B). TLR3 and dsRNA is shown in cartoon representation. The interaction residues are shown as a stick model. The important residues for stabilization of the complex are shown in bold. Hydrogen bonds identified using PyMOL shown in green (main color code: TLR3 (chain A), white; TLR3* (chain B), pale cyan; dsRNA, orange).

**Figure 5:**
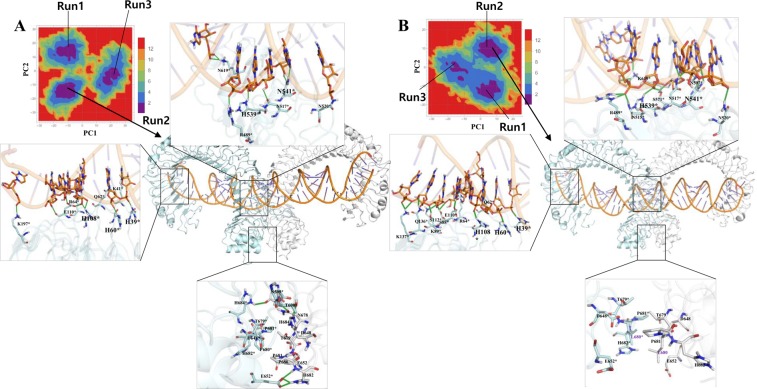
Hydrogen bond interactions between dsRNA and TLR3 mutants. Hydrogen bond interactions between dsRNA and TLR3 are shown. Representative structures were extracted based on the free-energy landscape of PC1 against PC2. (**A**) dTLR3^L412F^-dsRNA. (**B**) dTLR3^P680L^-dsRNA. Hydrogen bond interactions between dsRNA and TLR3* (chain B). TLR3 and dsRNA are shown in cartoon representation. The interaction residues are shown as a stick model. The important residues for stabilization of the complex are shown in bold. Hydrogen bonds identified using PyMOL are shown in green (main color code: TLR3 (chain A), white; TLR3* (chain B), pale cyan; dsRNA, orange).

**Figure 6:**
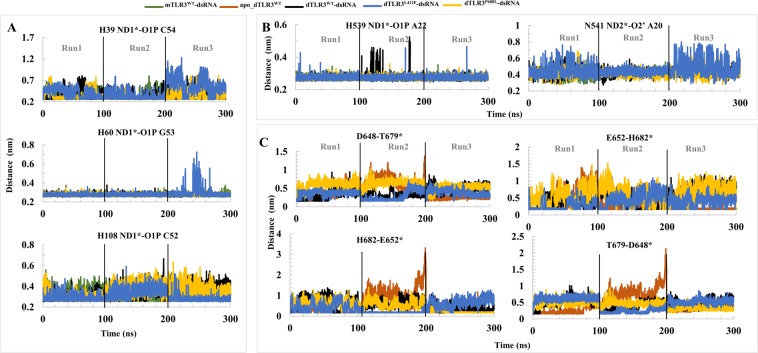
The minimum distance between crucial residues from TLR3 and dsRNA. (**A**) Minimum distance between TLR3 N-terminal site residues H39, H60, and H108 with dsRNA. (**B**) Minimum distance between TLR3 C-terminal site residues H539 and N541 with dsRNA. (**C**) Minimum distance between TLR3 dimer interface residues. The distances for TLR3* (chain B) and dsRNA (strand 1 (1–46) strand 2 (47–92)) are shown.

To evaluate the intermolecular interactions between dsRNA and TLR3 within all of the complexes, we considered the representative structures from a FEL analysis of PC1 and PC2. The FEL plot for all of the complexes showed no major transition states during last 60 ns (3 runs from each complex) indicating that TLRs may not undergo large conformational changes in the ECD upon ligand binding (Figs 4 and 5). However, ligands may induce minor conformational changes in the ECD, providing a dimerization interface with a symmetrical arrangement at TIR domains to recruit adaptor proteins and for subsequent signaling. From the representative structures, we observed that the TLR3 amino acids exhibiting conservation among species, such as His39, His60, and His108, at the N-terminal site, form hydrogen bonds with consecutive phosphate groups from the backbone of the dsRNA, whereas at the C-terminal site, His539 and Asn541 form hydrogen bonds with the phosphate group and sugar molecule, respectively (Figs 4 and 5). To analyze the consistency of these interactions at the N- and C-terminal sites, we examined the minimum distances for the entire MD trajectories of all of the complexes from TLR3* (chain B) (Fig. 6). Among 3 conserved amino acids, the interaction between His60 and dsRNA showed a stable hydrogen bond throughout the simulation in all of the complexes. The distances from His39 and His108 to dsRNA showed large fluctuations during the simulations in all of the complexes. In particular, the distance between His108 and dsRNA was stable compared to that between His39 and dsRNA in dTLR3^WT^-dsRNA complex. This suggests that His60 and His108 are needed to stabilize the TLR3-dsRNA complex. Surprisingly, His39 showed less consistent hydrogen bonding with dsRNA in all of the complexes. However, slight fluctuations were observed for wild-type than the mutant complexes. We assume that this interaction is also crucial for stabilizing the complex, consistent with previous findings indicating that a His39 mutation results in a loss of TLR3 signaling activity. However, mutations at His60 and His108 partially disrupted TLR3 signaling^24^. Therefore, our results support the complementary roles of His39, His60, and His108 interactions with dsRNA. Apart from these conserved residues, several other hydrogen bonds were observed between TLR3 and dsRNA (Figs 4 and 5). Moreover, the similar interactions were observed for TLR3 (chain A) in the N-terminal site (Supplementary Fig. S4). Furthermore, we checked the interaction distances at the C-terminal site for His539 and Asn541 with dsRNA. His539 showed hydrogen bonding during simulations over time in all of the complexes, which is crucial for the stability of the C-terminal site of the complexes. The other crucial residue, Asn541 shows hydrogen bonding with the base of A20 from dsRNA in our representative structure, however it is reported that, this residue also has an interaction with O2′ from sugar molecule in the crystal structure. Hence, we checked the distance between ND2 from Asn541 with O2′ of sugar molecule from A20, we observed that this distance was flexible during simulations (Fig. 6). Based on the above analysis, we propose that His60 and His108 at the N-terminal site and His539 and Asn541 at the C-terminal site are likely to be important for the stability of the TLR3-dsRNA complex. Moreover, we assume that the conserved amino acid residue, His39, at the N-terminal site complementarily supports the stability of the TLR3-dsRNA complex.

### TLR3 ECD dimerization interface at the C-terminal site

**Figure 7:**
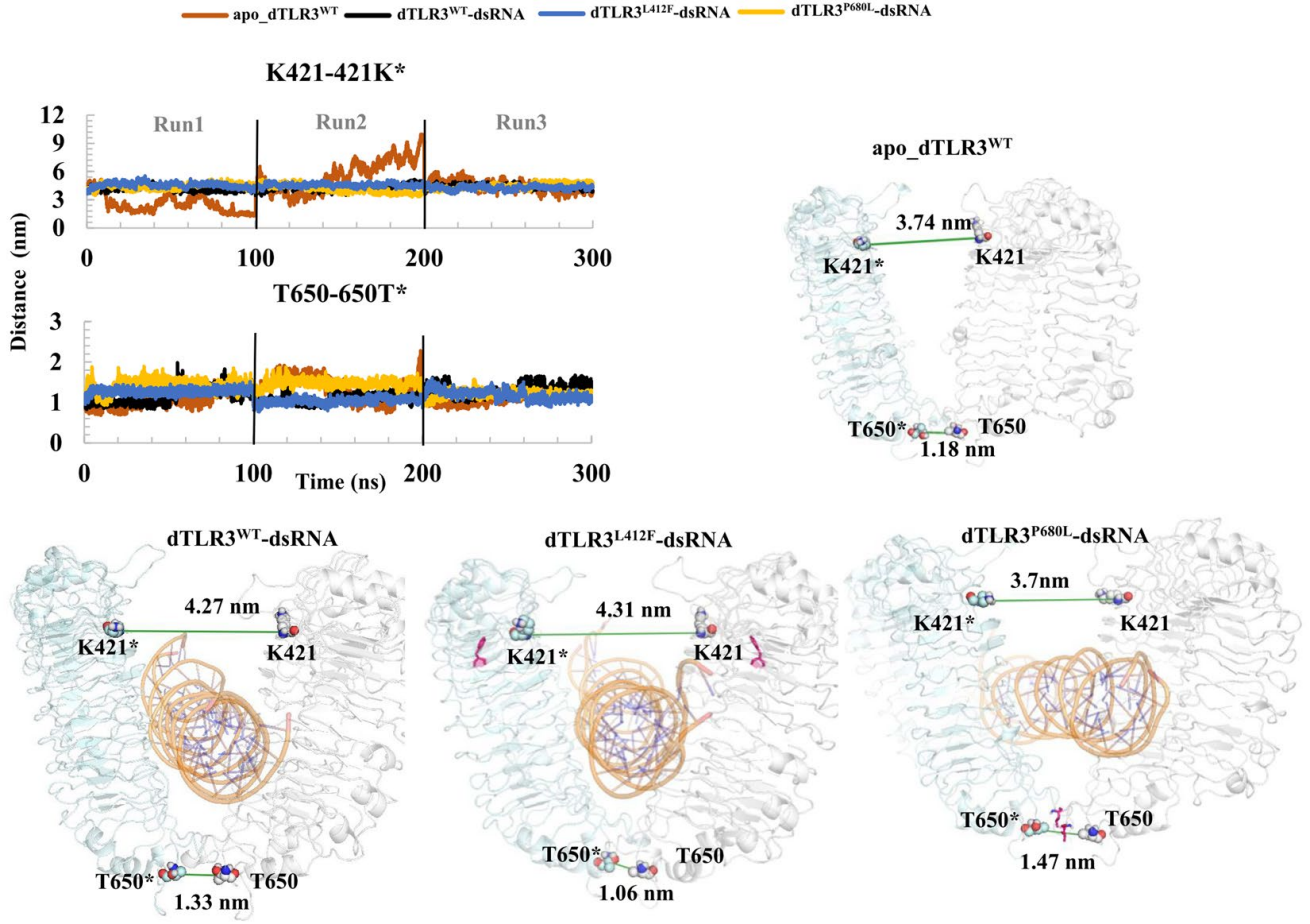
Distance between the individual TLR3 monomers. The distance between TLR3 monomers was calculated using K421 in the central region and T650 at the C-terminal site from both TLR3 chains and plotted for the MD trajectory of complexes (top left corner). Distance were mapped on the individual TLR3-dsRNA complexes (distance was calculated using center of mass (COM) among group of atoms of K421 and T650). K421 and T650 are shown as spheres and the line in green represents the distance. The mutant residues are shown in magenta.

Previous studies have reported that the TLR3 C-terminal dimerization site is crucial for ligand binding as well as for TLR3 signaling activity^24^. From our analysis we observed a weaker interface at the TLR3 ECD C-terminal dimerization site. In dTLR3^WT^-dsRNA and dTLR3^L412F^-dsRNA, we observed interface residues with E652/H682* from both the TLR3 monomers, indicating that upon ligand binding, this interaction pair is crucial. Apart from this interaction, we also observed few more interactions in dTLR3^L412F^-dsRNA complex (Fig. 5). However, in dTLR3^P680L^-dsRNA, we did not observe any hydrogen bond pairs in our representative structure, suggesting that this P680 is important at the TLR3 ECD C-terminus for holding the interface residues. Furthermore, in apo_dTLR3^WT^, we identified around 2 hydrogen bond pairs, i.e. His682/Glu652* and Glu652/His682* at the C-terminal site (Fig. 4). In addition, we calculated the distance between TLR3 monomers with representative Lys421 and Lys421* located at the central region of TLR3, which is exposed to solvent molecules (Fig. 7). We observed that the distance between the TLR3 monomers were largely varied from 3 simulation runs suggesting that apo_dTLR3^WT^ complex may not be stable in physiological conditions. The distance was higher in dTLR3^L412F^-dsRNA and lower in dTLR3^P680L^-dsRNA than in the wild-type complex (dTLR3^WT^-dsRNA). At the ECD C-terminal site, the distance between the monomer was calculated using Thr650 and Thr650* and found to be larger in the dTLR3^P680L^-dsRNA complex compared to other complexes at least in 2 simulation runs out of 3 runs (Fig. 7), indicates that P680L may influence the dimer interface. These results strongly suggest that slight deviations in the orientation between two individual chains due to variation in the internal motions of the mutant complexes may lead to an improper symmetrical orientation at the TIR-TIR domain level, which may explain the reduced TLR3 signaling activity from the mutant complexes.

### Interaction energies

**Figure 8:**
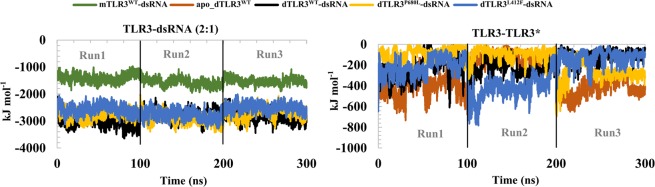
Interaction energy between the components of the TLR3 complexes. The interaction energy (summation of Coulomb and Lennard jones interaction terms) were calculated for components in TLR3 complex.

We calculated the interaction energy between the components within the TLR3 complexes (Fig. 8 and Table 1). The average interaction energy between dsRNA and TLR3 from 3 simulations of each complex were about −2873 kJ mol^−1^ for dTLR3^WT^-dsRNA, −2612 kJ mol^−1^ for dTLR3^L412F^-dsRNA and −2775 kJ mol^−1^ for dTLR3^P680L^-dsRNA indicate a lower binding affinity between dsRNA and TLR3 in mutant complexes compared to wild-type TLR3. The interaction energy of the dTLR3^WT^-dsRNA complex at pH 5.0 (Table 1 and Supplementary Fig. S5) is about -2602 kJ mol^−1^, which is slightly lower than dTLR3^WT^-dsRNA at pH 6.0, indicating that the affinity of dsRNA varies largely between pH 5.5 and 6.5 compared to pH 5.0, consistent with previous experimental reports^24^. In addition, we also verified the interaction energy between TLR3 monomers in the complexes, which were about −320 kJ mol^−1^ for apo_dTLR3^WT^, −188 kJ mol^−1^ for dTLR3^WT^-dsRNA, −259 kJ mol^−1^ for dTLR3^L412F^-dsRNA, and −153 kJ mol^−1^ for dTLR3^P680L^-dsRNA. The large interaction energy maintained between TLR3 monomers in apo_dTLR3^WT^ indicates that lower possibility of dsRNA binding in the pre-formed TLR3 ECD dimer complex. Similar interaction energies between TLR3 monomers were observed for dTLR3^WT^-dsRNA and dTLR3^L412F^-dsRNA in at least 2 simulations out of 3 runs (Table 1). However, the other mutant (dTLR3^P680L^-dsRNA) complex show a lower interaction energy than the wild-type complex in 2 simulations out of 3 runs (Table 1), indicating that the P680L mutation may influence the TLR3 C-terminal dimer interface which is required for effective dsRNA binding. Moreover, the observed interaction energies at the TLR3 ECD N-terminal-site between His39, His60, and His108 and C-terminal site His539 and Asn541 (from both the chains of TLR3) with dsRNA suggest that, although the distance fluctuates, between these residues and backbone of dsRNA (Fig. 6), the interaction energies were maintained, indicating that these residues are crucial for stabilization in the TLR3-dsRNA complex, consistent with previous reports^24^. In particular, His39 from dTLR3^WT^-dsRNA maintained their interaction energies with dsRNA, suggesting that H39 is also important for the stabilization of the TLR3-dsRNA complex^24^. The interaction energies at the C-terminal dimer interface also suggest that a weak dimer-interface at the TLR3 ECD C-terminal site is important for signal transmission (Table 1).

**Table 1:** The interaction energy given for all the TLR3 complexes.

Components	mTLR3^WT^-dsRNA (kJ mol^-1^)			apo_dTLR3^WT^ (kJ mol^-1^)			dTLR3^WT^-dsRNA (kJ mol^-1^)			dTLR3^L412F^-dsRNA (kJ mol^-1^)			dTLR3^P680L^-dsRNA (kJ mol^-1^)			dTLR3^WT^-dsRNA (at pH 5.0) (kJ mol^-1^)
	Run1	Run2	Run3	Run1	Run2	Run3	Run1	Run2	Run3	Run1	Run2	Run3	Run1	Run2	Run3	Run1
TLR3-dsRNA (2:1)	−1400.74 (38)	−1564.52 (42)	−1519.13 (28)				−3018.54 (48)	−2764.36 (39)	−2839.09 (42)	−2568.06 (36)	−2774.19 (35)	−2494.65 (19)	−2732.96 (39)	−2909.61 (47)	−2686.91 (42)	−2602.07 (48)
TLR3-TLR3*				−395.67 (19)	−155.94 (19)	−410.12 (30)	−216.42 (36)	−231.71 (14)	−118.93 (8.6)	−224.27 (27)	−422.65 (39)	−132.20 (10)	−92.7 (15)	−97.15 (4.4)	−271.42 (26)	−133.26 (28)
TLR3-dsRNA							−1569.05 (35)	−1275.26 (29)	−1300 (22)	−1350.54 (13)	−1370.43 (39)	−1361.67 (44)	−1353.62 (16)	−1410.75 (20)	−1413.02 (25)	−1123.21 (58)
TLR3*-dsRNA							−1449.49 (24)	−1489.1 (18)	−1538.89 (30)	−1217.52 (46)	−1403.76 (12)	−1132.99 (54)	−1379.34 (32)	−1498.88 (40)	−1273.89 (24)	−1478.86 (30)
H39-dsRNA							−97.46 (0.8)	−75.46 (8)	−99.12 (3.8)	−99.50 (1.7)	−83.71 (2.8)	−80.56 (9.7)	−101.76 (0.84)	−103.59 (2.4)	−96.49 (2)	−82.17 (3.6)
H39*-dsRNA	−98.11 (7.6)	−95.09 (2.2)	−112.55 (7)				−76.29 (7.8)	−89.78 (3.7)	−65.28 (2.6)	−97.16 (12)	−83.75 (3.1)	−28.96 (8.2)	−91.19 (6)	−99.15 (1.6)	−66.37 (3.7)	−76.57 (5.8)
H60-dsRNA							−83.29 (0.56)	−87.26 (0.71)	−86.14 (2.2)	−83.22 (0.4)	−86.94 (0.27)	−82.46 (0.25)	−86.41 (0.54)	−86.58 (0.63)	−85.12 (0.57)	−87.36 (0.21)
H60*-dsRNA	−88.00 (0.53)	−87.58 (0.62)	−91.49 (2.1)				−87.72 (0.6)	−86.78 (0.49)	−86.18 (0.49)	−111.95 (11)	−85.92 (0.2)	−83.38 (7.3)	−87.94 (0.89)	−86.47 (0.52)	−86.04 (0.41)	−87.69 (0.28)
H108-dsRNA							−88.65 (0.95)	−76.30 (7.7)	−81.65 (1.3)	−87.27 (0.59)	−84.28 (1.9)	−73.62 (6.4)	−88.84 (2)	−88.12 (1.3)	−92.45 (2.5)	−86.17 (0.82)
H108*-dsRNA	−79.83 (2.7)	−88.60 (1.2)	−91.03 (1.3)				−85.78 (0.59)	−78.49 (1.9)	−69.52 (1.9)	−91.56 (0.83)	−80.34 (0.29)	−98.01 (2.2)	−90.60 (1.5)	−65.93 (1.4)	−75.82 (4.1)	−82.73 (0.86)
H539-dsRNA							−96.05 (1.2)	−93.53 (0.69)	−89.15 (0.71)	−91.47 (1.2)	−96.74 (0.45)	−95.48 (0.9)	−94.41 (2.1)	−98.26 (0.45)	−97.83 (1.2)	−91.05 (1.2)
H539*-dsRNA	−90.16 (0.79)	−96.05 (0.81)	−99.81 (0.27)				−101.72 (1.1)	−93.2.0 (1.4)	−96.85 (0.21)	−89.80 (0.78)	−89.32 (0.6)	−93.84 (1)	−91.89 (1.1)	−98.36 (0.97)	−90.25 (1.8)	−98.78 (0.59)
N541-dsRNA							−69.27 (0.47)	−69.43 (0.85)	−41.34 (5.1)	−50.76 (6.7)	−71.86 (1.9)	−67.14 (0.61)	−68.25 (1.9)	−64.37 (1.5)	−62.60 (1.4)	−51.34 (4.4)
N541*-dsRNA	−49.56 (4.1)	−62.70 (1.5)	−58.36 (0.63)				−55.44 (4.4)	−56.28 (1.3)	−62.90 (0.94)	−50.50 (3.1)	−64.95 (0.83)	−35.32 (2.7)	−55.47 (2.5)	−59.66 (1.2)	−75.82 (4.2)	−57.52 (1.3)
D648-T679*				−21.36 (12)	−1.60 (0.96)	−42.45 (1)	−20.00 (8.1)	−7.17 (0.68)	−2.05 (0.75)	−7.28 (2)	−30.28 (9.2)	−4.58 (1.4)	−1.10 (0.83)	−0.52 (0.32)	−2.39 (2.1)	−3.46 (1)
E652-H682*				−22.48 (11)	−51.98 (19)	−128.17 (3.9)	−23.02 (7.2)	−61.72 (26)	−6.21 (4.7)	−83.46 (15)	−23.04 (14)	−24.15 (5.7)	−20.53 (9.6)	−15.35 (4.2)	−6.66 (5.2)	−32.90 (20)
T679-D648*				−34.03 (8.2)	−1.07 (1)	−5.59 (1.7)	−6.28 (4.8)	−11.38 (8)	−2.21 (0.81)	−0.53 (0.3)	−30.47 (7.1)	−0.1 (0.19)	−1.08 (0.28)	−1.84 (0.73)	−4.63 (0.47)	−1.85 (0.77)
H682-E652*				−74.99 (27)	−2.61 (2.1)	−73.31 (17)	−16.50 (14)	−5.23 (2.9)	−28.44 (8.7)	−41.22 (6.9)	−127.06 (5.3)	−7.25 (4.1)	−7.56 (2.2)	−10.58 (2.3)	−95.52 (24)	−2.56 (1.6)

Error estimation is given in brackets.

### Principal component analysis (PCA)

**Figure 9:**
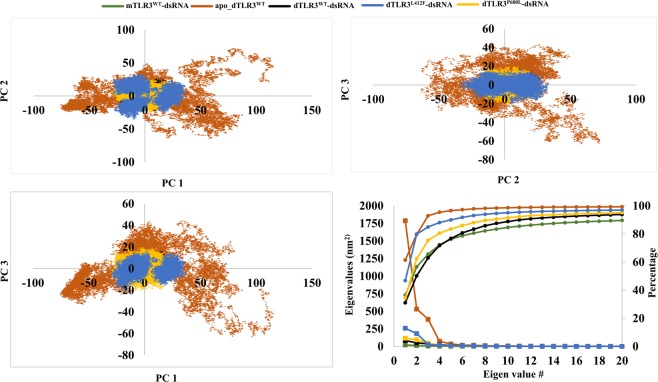
Projection of principal components onto the subspace. The projection of principal components extracted from MD trajectories of each TLR3 complex (backbone atoms) for the last 60 ns with 10 ps coordinates from 3 independent simulation runs. Principal components 1 and 2, Principal components 2 and 3, Principal components 1 and 3 are shown. Lines with square represent first 20 eigen vectors of covariance matrix. Lines with sphere represents the cumulative percentage of variance.

**Figure 10:**
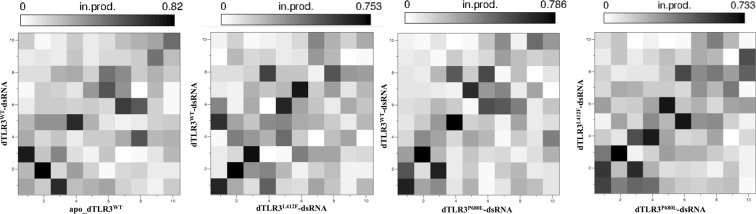
The overlap of covariance matrices between TLR3 complexes. The first 10 eigenvectors were selected for the overlap of covariance matrix between TLR3 complexes. Each block denotes the overlap of 2 eigenvectors from TLR3 complexes.

To understand the variations in the global motions, particularly those induced by mutations in the TLR3 complexes, we examined the concatenated MD trajectories using PCA for the last 60 ns from 3 simulations of each complex with 10-ps coordinates. The MD trajectories of the TLR3 complex systems indicate that the first 10 principal components contribute largely to the collective motions. The cumulative percentages of variance in motion obtained from the first 10 principal components were 84%, 98%, 88%, 95%, and 91% for mTLR3^WT^-dsRNA, apo_dTLR3^WT^, dTLR3^WT^-dsRNA, dTLR3^L412F^-dsRNA, and dTLR3^P680L^-dsRNA, respectively. The projections of eigenvectors 1 and 2, 2 and 3, and 1 and 3 are shown in Fig. 9, which clearly indicate that the first 2 principal components of all MD trajectories exhibited periodic jumps with a small energy barrier except apo_dTLR3^WT^. particularly, the dTLR3^L412F^-dsRNA complex showed 3 conformational states in the subspace form 3 simulation runs. However, other dsRNA bound complexes (mTLR3^WT^-dsRNA, dTLR3^WT^-dsRNA and dTLR3^P680L^-dsRNA) showed that, sampling from 3 simulation runs were occupied the same basin in the phase space. Furthermore, the diagnolized covariance matrix for the back-bone atoms of the TLR3 complex was 59.25 nm^2^ for mTLR3^WT^-dsRNA, 2911.09 nm^2^ for apo_dTLR3^WT^, 268.2 nm^2^ dTLR3^WT^-dsRNA, 552.48 nm^2^ for dTLR3^L412F^-dsRNA, and 339.35 nm^2^ for dTLR3^P680L^-dsRNA. Further, we verified the overlap between the covariance matrices of the four (apo_dTLR3^WT^, dTLR3^WT^-dsRNA, dTLR3^L412F^-dsRNA and dTLR3^P680L^-dsRNA) dimer complexes. For this, we used the first 10 eigen vectors, which contributes more than 80% of the overall variance in the TLR3 complexes. dsRNA-bound wild-type and mutant TLR3 complexes were compared to each other. Over all the wild-type and mutant complexes did not share substantial overlap, i.e. for apo_dTLR3^WT^ vs dTLR3^WT^-dsRNA (normalized overlap of 0.21), for dTLR3^WT^-dsRNA vs dTLR3^L412F^-dsRNA (normalized overlap of 0.44), for dTLR3^WT^-dsRNA vs dTLR3^P680L^-dsRNA (normalized overlap of 0.57), for dTLR3^L412F^-dsRNA vs dTLR3^P680L^-dsRNA (normalized overlap of 0.49). However, root means square inner product (RMSIP) plot shows that there are several similarities between distinct eigen vector of different rank indicate that the global dynamics of the wild type and mutant bound complexes are not largely different. In particular, RMSIP of the first three eigen vectors between dTLR3^WT^-dsRNA and dTLR3^L412F^-dsRNA shows significant overlap (Fig. 10). Since the large magnitude was represented by first three eigenvectors, in the wild-type and mutant TLR3 complex, we used porcupine plots to explain the direction of motions of the mode of the first 3 eigenvectors (Supplementary Fig. S6). The dTLR3^WT^-dsRNA and dTLR3^L412F^-dsRNA shows the individual chains of TLR3 ECD were facing opposite to each other whereas, in dTLR3^P680L^-dsRNA monomers undergo rotational motion towards each other in the first eigen vector. Furthermore, the second eigen vector for dTLR3^WT^-dsRNA and dTLR3^L412F^-dsRNA, monomers were facing opposite to each other with rotational motion, whereas in dTLR3^P680L^-dsRNA, monomers were facing towards each other. The third eigen vector shows that, though the slight variation in the motion was observed in the complexes, the magnitude of the direction of motion is lower compared to first 2 eigenvectors. The above results indicate that, global dynamics of wild-type and mutant TLR3 ECD complexes are not largely different, however, the observed local structural modifications at the dimer interface might play a crucial role to reorient the molecule for the proper signaling at the intracellular level.

### Network centrality analysis

**Figure 11:**
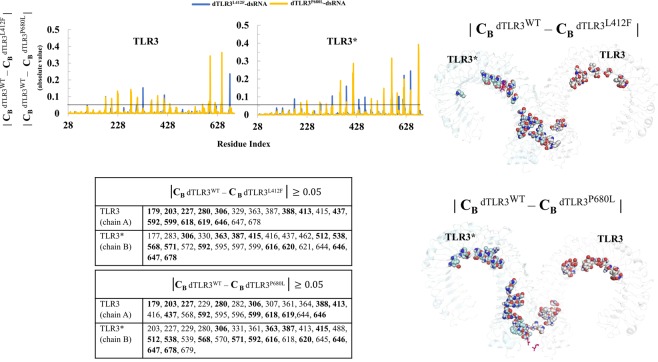
Variation in residue betweenness centrality (C_B_) values calculated for dsRNA bound wild-type (dTLR3^WT^-dsRNA) and mutant (dTLR3^L412F^-dsRNA, dTLR3^P680L^-dsRNA) TLR3 complexes. The plot shows the difference of residue betweenness centrality (C_B_) values (absolute value) for wild-type and mutant TLR3 complexes. Black line represents the cut-off (*C*_*B*_ ≥ 0.05) used to select functionally important residues. Residues selected based on the difference of C_B_ values were mapped using a sphere model on the TLR3 mutant complexes and also listed on the table. The common residues from both the mutants are highlighted bold on the table. Mutant residues are shown in magenta on the structures (main color code: TLR3 (chain A), white; TLR3* (chain B), pale cyan; dsRNA, orange). For clarity, the labeling of TLR3 complexes are mentioned without dsRNA.

We considered the representative structures of TLR3 wild-type (mTLR3^WT^-dsRNA, apo_dTLR3^WT^, dTLR3^WT^-dsRNA) and mutant (dTLR3^L412F^-dsRNA and dTLR3^P680L^-dsRNA) complexes from simulations and then used these to construct a network with a residue-to-residue (non-hydrogen atoms) cut-off distance of 0.7 nm. Using this network, we calculated the residue betweenness centrality (C_B_) value, which provides information flow for each residue (node) in the network to understand the global mediating nodes in the dsRNA bound TLR3 complexes. A node with a high C_B_ value may be associated with a functional role in signal transduction. Therefore, we selected the residues or nodes with *C*_*B*_ ≥ 0.1 and mapped these residues on the structure in order to understand the difference between the TLR3 wild-type and mutant complexes (Supplementary Fig. S7). In the dTLR3^WT^-dsRNA complex, we found that the residues propagated from central region to C-terminal site. Hence we assume that, central region to C-terminal site, might be crucial for signal transmission and provide the conformation shift required at the dimer interface upon dsRNA binding to TLR3. To our knowledge this is the first instance of such an observation being reported in TLR3. The variation in the position of residues were identified in the mutant complexes, however, similar distribution occurs like that of dTLR3^WT^-dsRNA (Supplementary Fig. S7). Further, using the condition (|**C**_**B**_ dTLR3^WT^ – **C**_**B**_ dTLR3^L412F^ | ≥ 0.05), (|**C**_**B**_ dTLR3^WT^ – **C**_**B**_ dTLR3^P680L^ | ≥ 0.05), we calculated the values (absolute value) with large variation between dTLR3^WT^-dsRNA and dTLR3^L412F^-dsRNA as well as dTLR3^WT^-dsRNA and dTLR3^P680L^-dsRNA (Fig. 11). From this analysis we observed that, ~50% of the residues are common form the mutants, which may influence the modification of the residue signal propagation compared to wild-type. Moreover, it is observed that the central to C-terminal regions are varied between complexes indicate that residues at these regions play a crucial role for the conformational changes in TLR3 wild-type and mutant complexes (Fig. 11). However, further biochemical or computational studies on full length TLR3 are needed to confirm the roles of these residues.

## Discussion

TLRs play a critical role in innate immunity by sensing molecular patterns from pathogens. TLR3 localizes on endosomes and senses dsRNA from pathogens at mild acidic pH (5.5 to 6.5), thereby triggering downstream signaling to induce immune responses. The structures of human and mouse TLR3 ECD have been obtained through protein crystallography^21,23^. The human TLR3 ECD is found in a monomeric state, whereas the mouse TLR3 structure is in a dsRNA-bound TLR3 dimeric state where the major interaction site for the dsRNA and C-terminal dimer interface is found in the TLR3 ectodomain. It was previously suggested that for human TLR3, C-terminal dimerization at the ECD is required for ligand binding^24^. In *TLR3*, L412F polymorphism is associated with several human diseases. Despite the availability of structural information regarding the TLR3 ECD, the dynamic behavior of the human TLR3-dsRNA complex, as well as the structural and functional relationship between wild-type and polymorphic (L412F) TLR3, have not yet been determined. As such, in this study, we examined the dynamic behavior of the TLR3-dsRNA complex. In particular, we designed 5 different complex systems (mTLR3^WT^-dsRNA, apo_dTLR3^WT^, dTLR3^WT^-dsRNA, dTLR3^L412F^-dsRNA, and dTLR3^P680L^-dsRNA) and evaluated these complexes using MD simulations. For the mTLR3^WT^-dsRNA complex, a single TLR3 monomer was found to bind to dsRNA at N- and C-terminal sites, and there were similar hydrogen bonds on average, compared to the dTLR3^WT^-dsRNA complex for a single monomer with dsRNA (Fig. 3). Despite there being similar hydrogen bonds, a single monomer cannot initiate downstream signaling and several reports have suggested that cooperative binding between ligand and two TLR monomers (2:1 complex) stabilizes the whole complex^23,45^. Unlike other TLRs, endosomal TLRs may exist as pre-formed dimers, as suggested for human TLR9^25^, and TLR3 ECD dimerization is required for effective ligand binding^24^. In our simulations, apo_dTLR3^WT^ (dsRNA-unbound TLR3-ECD dimer) showed large RMSD fluctuations compared to dsRNA bound TLR3 complexes, which is similar to the previous results obtained for apo TLR4 dimer^27^. However, the MD-2 binds to the concave region of TLR4 and acts as co-receptor whereas dsRNA binds to TLR3 at the N-terminal (LRR-NT and LRR1-3) and C-terminal (LRR 19–21) site of individual TLR3 monomers in the lateral convex surface. Moreover, unlike other TLRs, which exhibits homo or hetero dimerization at the various regions of C-terminal domain, dimerization interface for TLR3 is located at LRR-CT. The distance between the C-termini from two monomers of other TLRs^45^ is large compared to 0.7 nm distance observed for TLR3^23^. Hence, suggest that despite sharing common fold with other TLRs, TLR3 may exhibit dissimilar conformational changes. Furthermore, apo_dTLR3^WT^ showed ECD dimer interface in the C-terminal region, as well as higher interaction energy compared to dTLR3^WT^-dsRNA (at least in 2 simulation runs). However, the distance between each monomer in apo_dTLR3^WT^ is largely varied in 3 simulation runs, indicating that pre-formed TLR3 dimers may not be stable in physiological conditions. Hence, we propose that TLR3 may exist as a monomer at least in the ECD, supporting observations that TLR3 exists as a monomer in solution^21^.

The variation in the interaction energies and hydrogen bonds identified for the mutants (dTLR3^L412F^-dsRNA and dTLR3^P680L^-dsRNA) compared to dTLR3^WT^-dsRNA complex suggests that these mutations induce certain conformational changes. The major interaction sites between TLR3 and dsRNA observed at the N-terminal and C-terminal site were consistent with previous reports^24^. During simulations, the interaction of H39 and N541 with the backbone of dsRNA showed large fluctuations in terms of distance, however, the interaction energy was maintained. These results suggest that His39, His60, and His108 from the N-terminal site and H539 and N541 from the C-terminal site are essential for dsRNA (Figs 4 and 5). We observed hydrogen bonding in one pair (E652/H682*) in our representative structures of dTLR3^WT^-dsRNA and dTLR3^L412F^-dsRNA, suggesting that along with the N- and C-terminal interactions between dsRNA and TLR3 ECD, a weak dimer interface at the ECD C-terminal between two TLR3 monomers is required for the cumulative binding effect between dsRNA and TLR3 at the extracellular level. However, we did not observe a dimer interface at the ECD C-terminal in dTLR3^P680L^-dsRNA complex in 2 simulation runs out of 3, strongly suggesting that Pro680 is crucial for maintaining the dimer interface for dsRNA binding in the wild-type TLR3-complex (Fig. 5). Moreover, the interaction energy between TLR3 monomers is higher in dTLR3^WT^-dsRNA and dTLR3^L412F^-dsRNA compared to dTLR3^P680L^-dsRNA complex (Fig. 8 and Table 1). This result is similar to the results of previous mutational studies, suggesting that TLR3 dimerization is required for dsRNA binding^24^. Furthermore, the TLR3 monomer distances in the complexes indicate a reduced binding affinity for dsRNA in the mutant complexes, such as a large distance for dTLR3^L412F^-dsRNA, which exhibits less cohesiveness between the monomers. However, the distance between the monomers at the C-terminal site is larger for dTLR3^P680L^-dsRNA compared to other complexes (in 2 simulation runs), due to local structural alternations as a result of being surrounded by the mutant residue (Fig. 7). The similar dominant motions observed for first three eigenvectors between dsRNA bound wild-type and mutant complexes, suggest that, the mutant complexes undergo local structural modifications, thereby altering the intrinsic dynamics and potentially leading to reduced TLR3 signaling activity. The network centrality analysis suggests that distribution of functionally-important residues in TLR3-dsRNA complex located from the central region to C-terminal site. Despite the similar distribution in mutant complexes compared to wild-type, the variant regions observed in mutant complexes, indicate that these regions induce conformational changes which affect dimerization at the C-terminal site, as well as having an impact on the symmetrical arrangement of the TIR-TIR domain in the cytoplasm. As a result, these mutations could impair TLR3 signaling.

In recent years, MD simulations have become a very powerful tool for understanding the intrinsic dynamic and conformational changes induced by ligands and mutations. In this study, our MD simulations were limited to a 100-ns timescale with 3 independent simulation runs; however, TLRs are massive receptor complexes that may require microsecond simulations to unravel the minute conformational changes that occur upon ligand binding at the ECD. Based on our results, we believe that an MD approach can extend our understanding of the dynamic behavior of TLR3, which may aid in the developing therapeutic strategies targeting TLR3.

## Supplementary information

Insights into the dynamic nature of the dsRNA-bound TLR3 complex
