# Supplementary material for: Insights into the dynamic nature of the dsRNA-bound TLR3 complex
Source: Sci Rep. 2019 Mar 6;9:3652. doi: 10.1038/s41598-019-39984-8 (PMC6403236; doi:10.1038/s41598-019-39984-8)
Supplement: Supplementary file 1 — Insights into the dynamic nature of the dsRNA-bound TLR3 complex [file 41598_2019_39984_MOESM1_ESM.docx]

**Supplementary information**

**Insights into the dynamic nature of the dsRNA-bound TLR3 complex**

**Vijayakumar Gosu^1^, Seungwoo Son^1^, Donghyun Shin^1*^ and Ki-Duk Song^1,2^**^*^

**^1^**Department of Animal Biotechnology, Chonbuk National University, Jeonju 54896, Republic of Korea

**^2^**The Animal Molecular Genetics and Breeding Center, Chonbuk National University, Jeonju 54896, Republic of Korea

***Correspondence**

Donghyun Shin

Email: [sdh1214@gmail.com](file:///C:\Users\SONGKD\OneDrive%20-%20전북대학교\MS\TLR-Dr%20Gosu\sdh1214@gmail.com)

Ki-Duk Song

Tel: +82-63-219-5523, Email: [kiduk.song@gmail.com](mailto:kiduk.song@gmail.com)

**
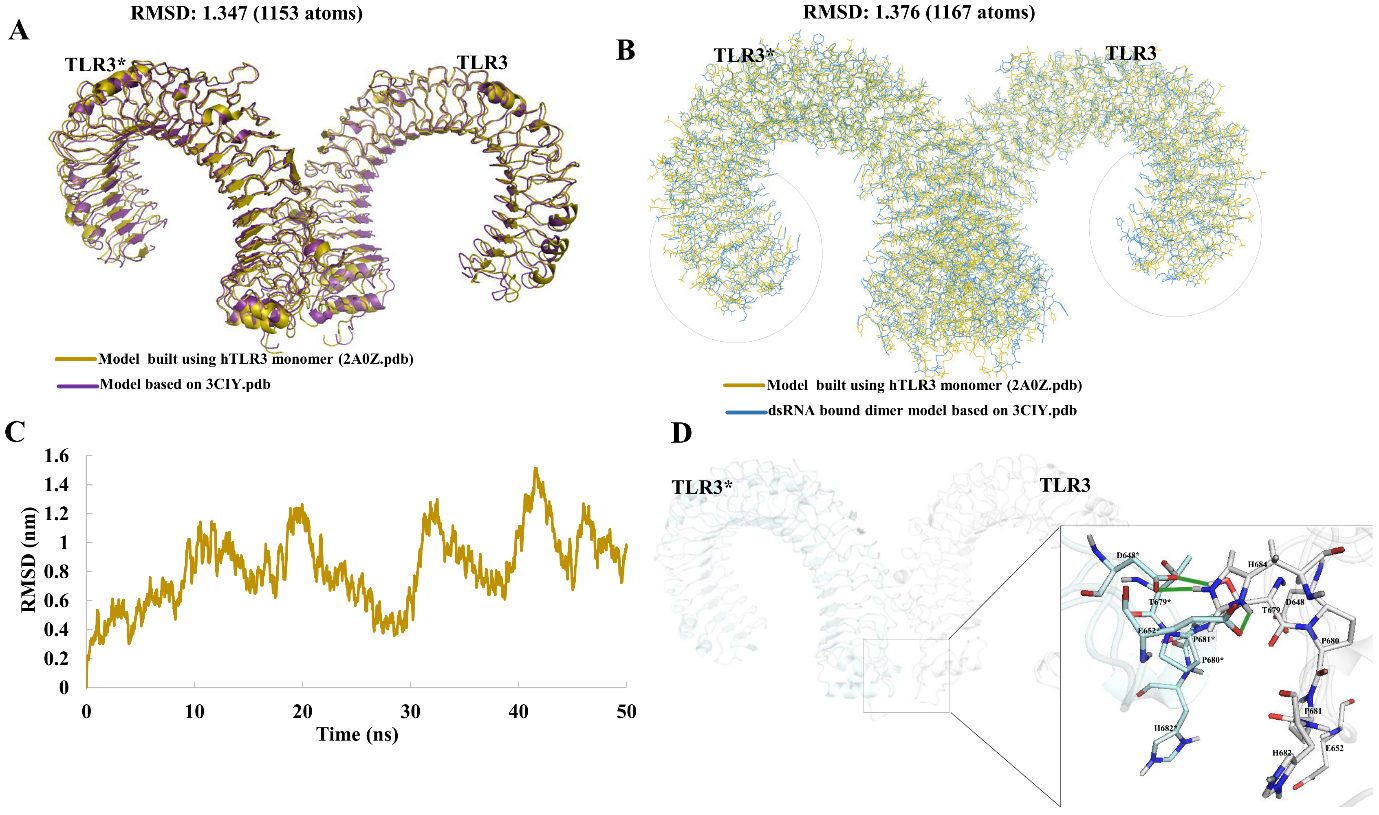
**

**Supplementary Figure S1: RMSD of apo-TLR3 dimer built using human TLR3 monomer structure.** (A) Superimposition of apo-TLR3 dimer built using human TLR3 monomer structure and apo_dTLR3^WT^. (B) Apo-TLR3 dimer built using human TLR3 monomer structure and dTLR3^WT^-dsRNA (for clarity we have removed dsRNA). (C) The RMSD of the 50 ns MD trajectory of the apo-TLR3 dimer is similar to that of apo_dTLR3^WT^. (D) Similar hydrogen bond interactions were identified in the low energy structure compared to apo_dTLR3^WT^.


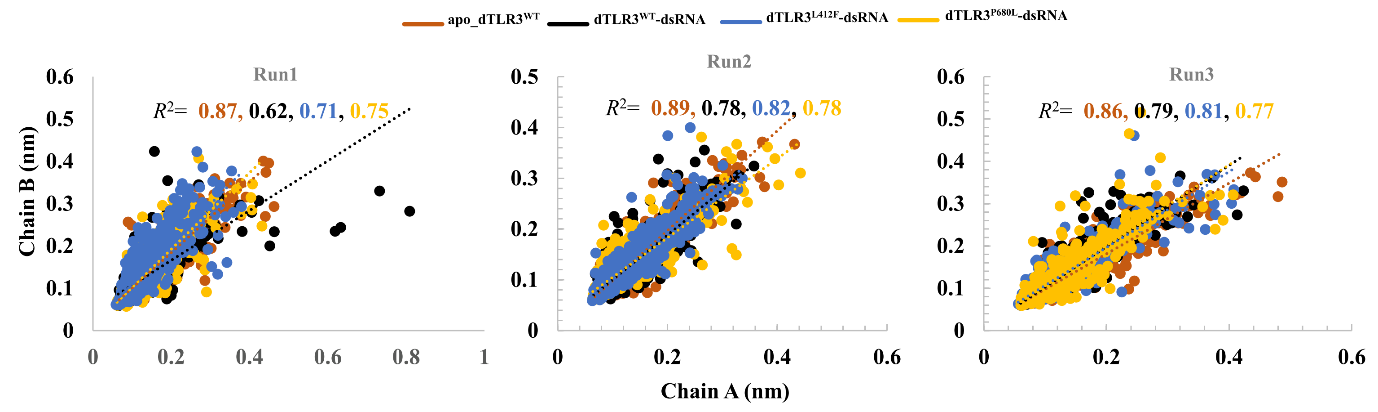


**Supplementary Figure S2:**  TLR3 (chain A) backbone atoms compared to TLR3* (chain B) to analyze the divergence or cohesiveness within the complexes. The linear trend line has been drawn to highlight the divergence in TLR3.


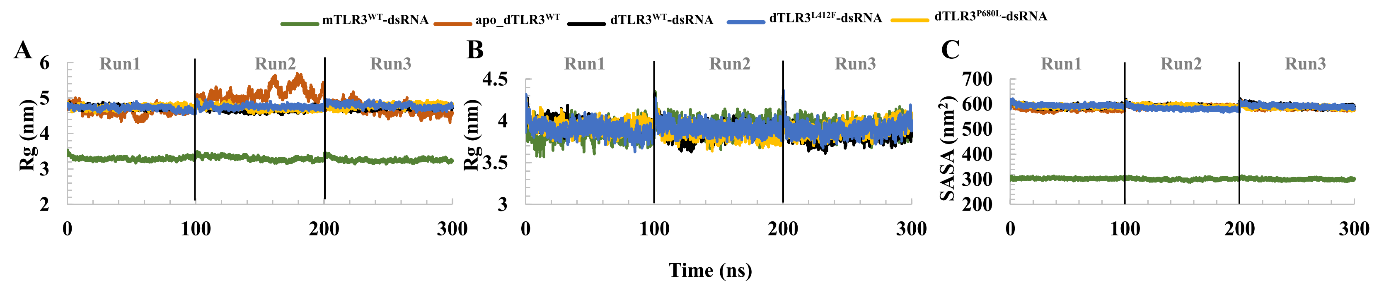


**Supplementary Figure S3: Radius of gyration (Rg) and solvent accessible surface area (SASA). (**A) Rg of backbone atoms of TLR3. (B) Rg of dsRNA. (C) SASA of the TLR3.


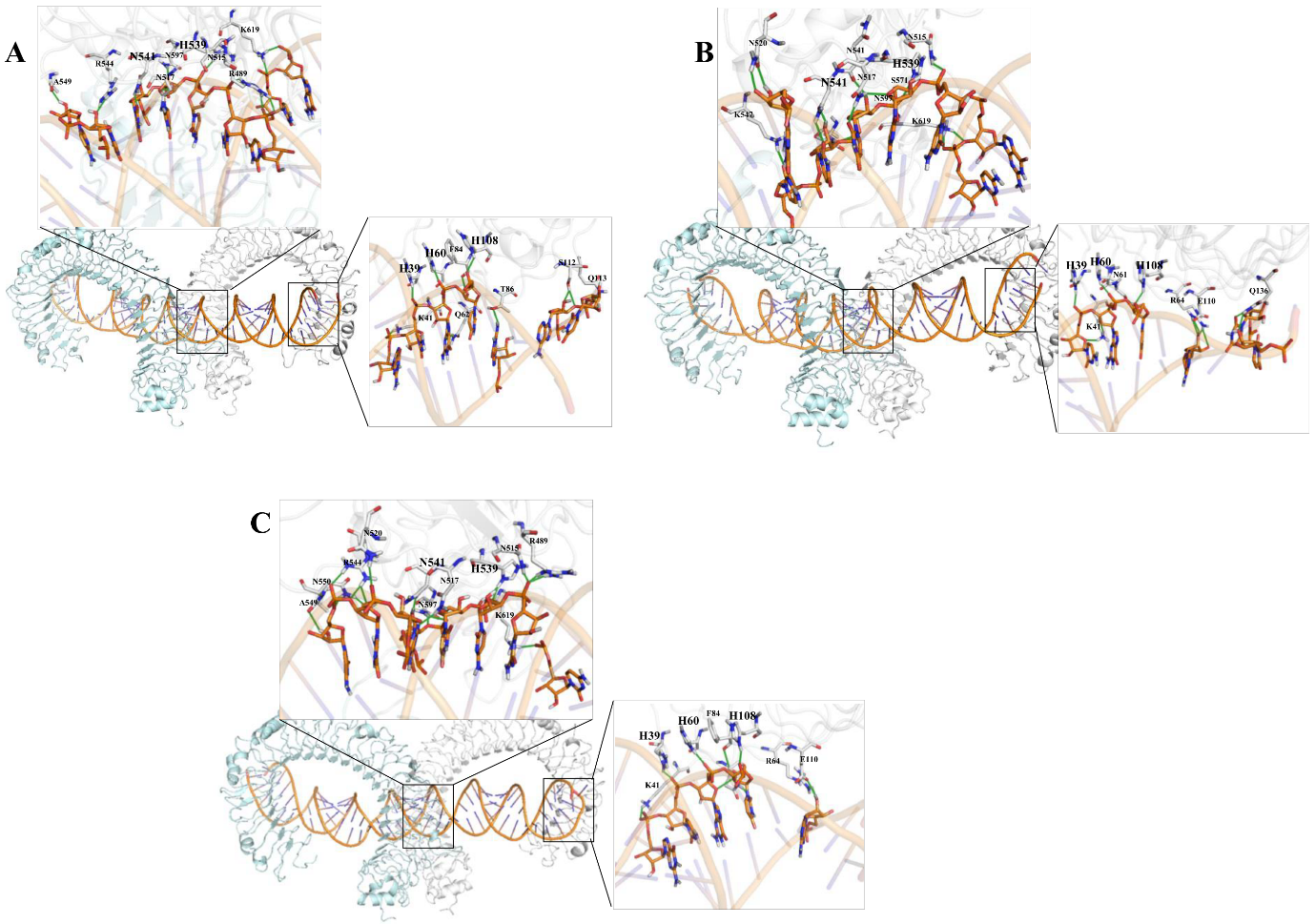


**Supplementary Figure S4: Hydrogen bond interaction between dsRNA and TLR3 complexes.** Hydrogen bond interactions between dsRNA and TLR3 (chain A) are shown. Representative structures were extracted based on the free-energy landscape of PC1 against PC2. (A) dTLR3^WT^-dsRNA. (B) dTLR3^L412F^-dsRNA. (C) dTLR3^P680L^-dsRNA. TLR3 and dsRNA is shown in cartoon representation. The interaction residues are shown as a stick model. The important residues for stabilization of the complex are shown in bold. Hydrogen bonds identified using PyMOL shown in green (main color code: TLR3 (chain A), white; TLR3* (chain B), pale cyan; dsRNA, orange).


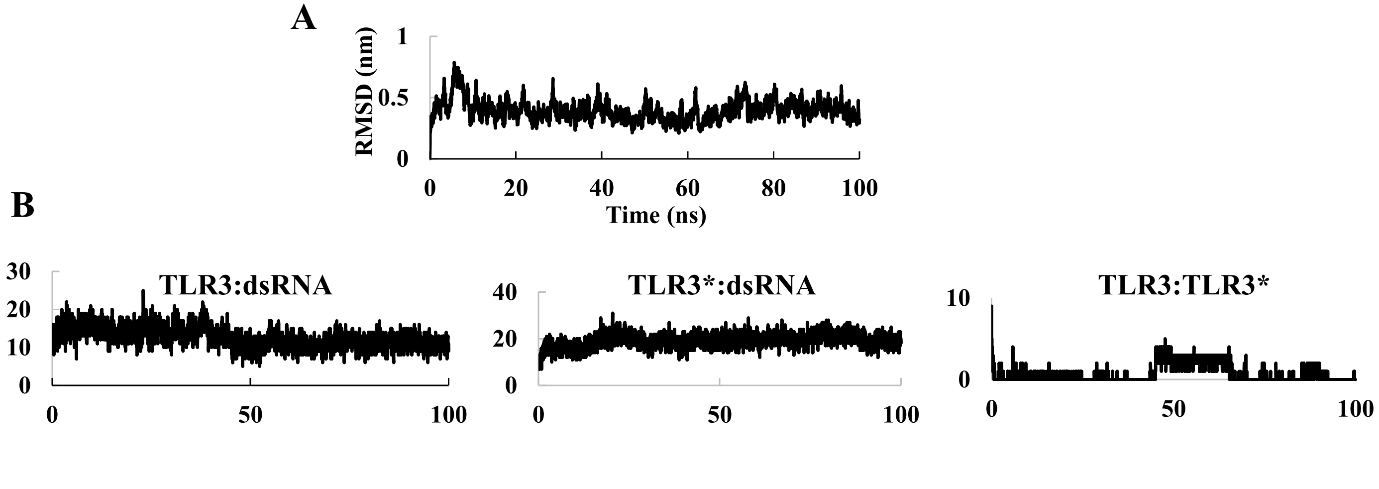


**Supplementary Figure S5: Dynamic parameters of TLR3 wild-type complex at pH 5.0.** (A) RMSD of TLR3 backbone atoms. (B) Number of hydrogen bonds between components in dTLR3^WT^-dsRNA.


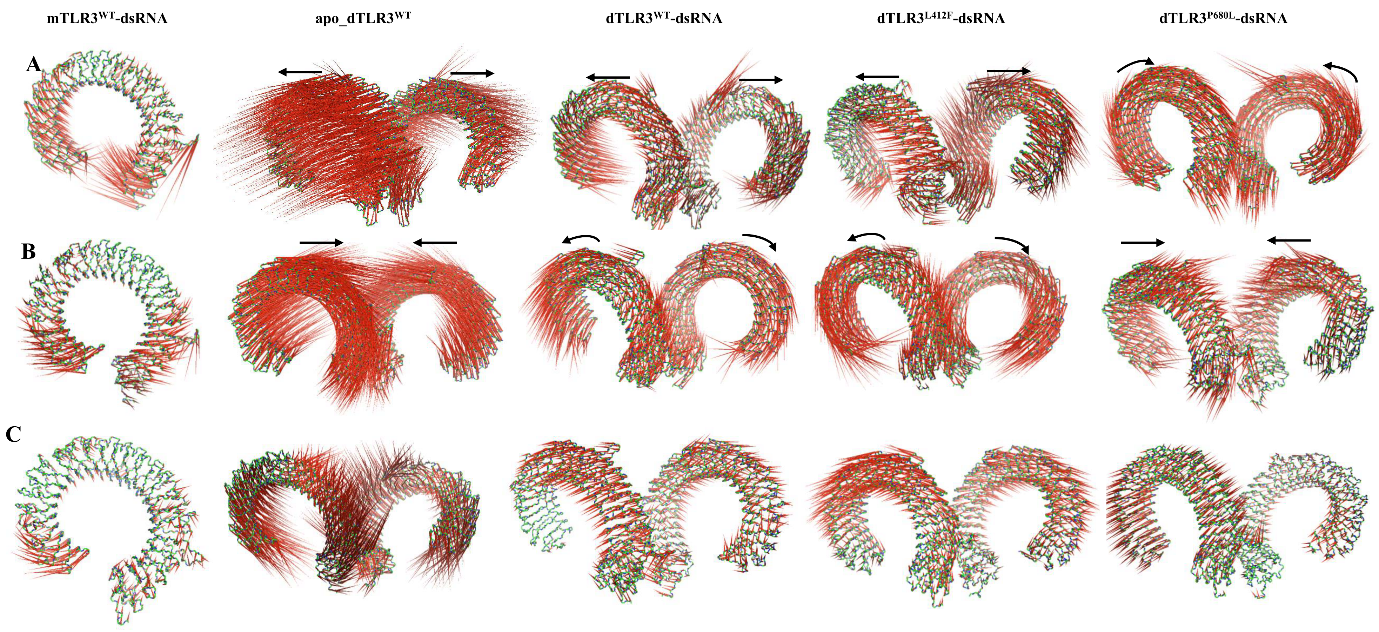


**Supplementary Figure S6: Porcupine plots of TLR3 complexes.** Porcupine plots were drawn using PyMOL to examine the direction of motions along eigenvector 1 (A), eigenvector 2 (B) and eigenvector 3 (C) for the last 60 ns of MD trajectory of the 3 independent simulations for all the TLR3 complexes using 30 frames generated between two extreme projections along trajectory.


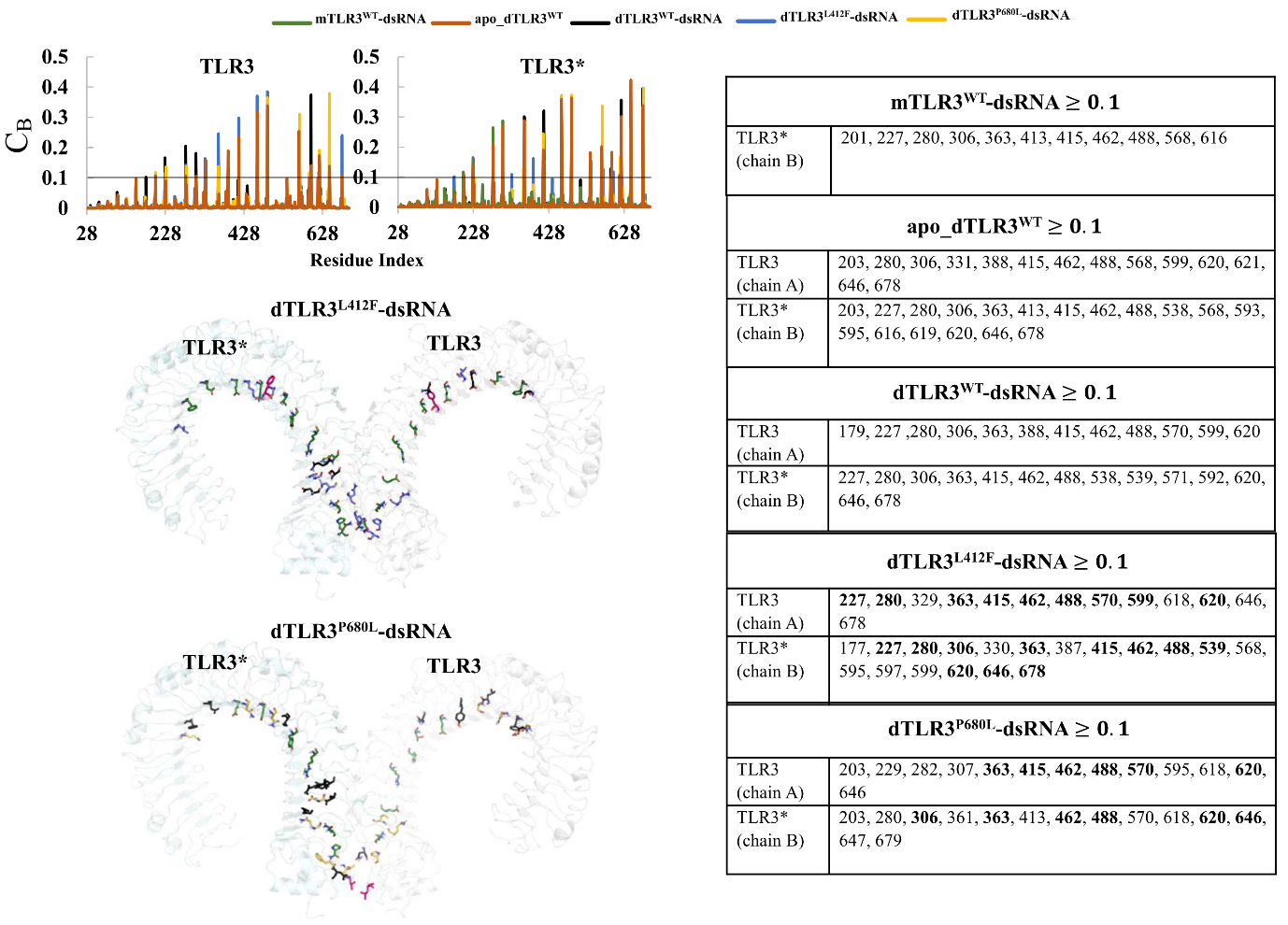


**Supplementary Figure S7: Residue betweenness centrality (C*_B_*).** The betweenness centrality was calculated using the representative structures of mTLR3^WT^-dsRNA, apo_dTLR3^WT^, dTLR3^WT^-dsRNA, dTLR3^L412F^-dsRNA, and dTLR3^P680L^-dsRNA. The plot (top left corner) shows the betweenness centrality of individual chains of the TLR3 complex. Pink line represents the cut-off (*C_B_*$\geq$0.1) used to select functionally important residues. Residues selected from the (*C_B_*$\geq$0.1) were mapped on the structures (dTLR3^L412F^-dsRNA and dTLR3^P680L^-dsRNA) using a stick model (black: residues from dTLR3^WT^-dsRNA, blue: residues from dTLR3^L412F^-dsRNA, yellow: residues from dTLR3^P680L^-dsRNA and green: common residues from wild-type and mutant complexes). Residues numbers (*C_B_*$\geq$0.1) are listed on the table for all the TLR3 complexes. Mutant residues are shown in magenta (main color code: TLR3 (chain A), white; TLR3* (chain B), pale cyan; dsRNA, orange).
